# Machine Learning of Allosteric Effects: The Analysis of Ligand-Induced Dynamics to Predict Functional Effects in TRAP1

**DOI:** 10.1021/acs.jpcb.0c09742

**Published:** 2020-12-28

**Authors:** Mariarosaria Ferraro, Elisabetta Moroni, Emiliano Ippoliti, Silvia Rinaldi, Carlos Sanchez-Martin, Andrea Rasola, Luca F. Pavarino, Giorgio Colombo

**Affiliations:** † Istituto di Scienze e Tecnologie Chimiche “Giulio Natta”− SCITEC, Via Mario Bianco 9, 20131 Milano, Italy; ‡ Institute for Advanced Simulation (IAS-5) and Institute of Neuroscience and Medicine (INM-9), Computational Biomedicine, Forschungszentrum Jülich, 52425 Jülich, Germany; § JARA-HPC, Forschungszentrum Jülich, D-54245 Jülich, Germany; ∥ Dipartimento di Scienze Biomediche, Università di Padova, viale G. Colombo 3, 35131 Padova, Italy; ⊥ Dipartimento di Matematica “F. Casorati”, Università di Pavia, Via Ferrata 5, 27100 Pavia Italy; # Dipartimento di Chimica, Università di Pavia, via Taramelli 12, 27100 Pavia, Italy

## Abstract

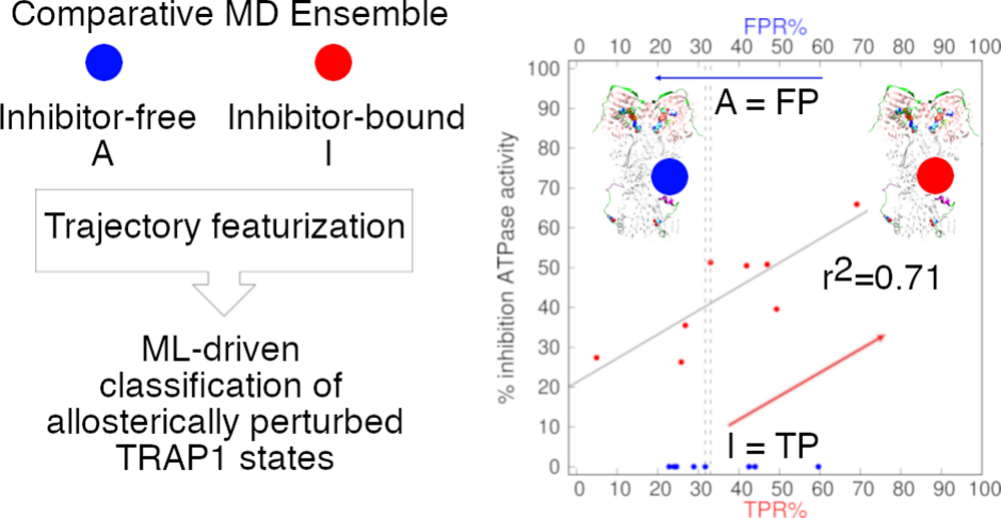

Allosteric molecules provide a powerful means to modulate protein function. However, the effect of such ligands on distal orthosteric sites cannot be easily described by classical docking methods. Here, we applied machine learning (ML) approaches to expose the links between local dynamic patterns and different degrees of allosteric inhibition of the ATPase function in the molecular chaperone TRAP1. We focused on 11 novel allosteric modulators with similar affinities to the target but with inhibitory efficacy between the 26.3 and 76%. Using a set of experimentally related local descriptors, ML enabled us to connect the molecular dynamics (MD) accessible to ligand-bound (perturbed) and unbound (unperturbed) systems to the degree of ATPase allosteric inhibition. The ML analysis of the comparative perturbed ensembles revealed a redistribution of dynamic states in the inhibitor-bound versus inhibitor-free systems following allosteric binding. Linear regression models were built to quantify the percentage of experimental variance explained by the predicted inhibitor-bound TRAP1 states. Our strategy provides a comparative MD–ML framework to infer allosteric ligand functionality. Alleviating the time scale issues which prevent the routine use of MD, a combination of MD and ML represents a promising strategy to support *in silico* mechanistic studies and drug design.

## Introduction

*In silico* hit-to-lead optimization is a challenging task in drug discovery. High attrition rates in virtual screening campaigns are associated with prioritization of hits with a predicted binding affinity that does not always match the expected efficacy *in vitro*/*vivo*.^1^ Determining a correlation between affinity and efficacy becomes even more challenging in the presence of allosteric compounds, as ligand effects at a distal site are often identified by monitoring substrate processing in the orthosteric pocket. In this respect, occurrence of “flat SAR” or “functional switches” as a consequence of even small changes in ligand structure points out how efficacy is not a mere function of affinity.^2^ Efficacy often depends on changes in system dynamics and kinetics. According to the conformational selection binding model proposed by Nussinov, ligands preferably bind to the best-matching protein conformation from an ensemble of states and shift the equilibrium toward that state.^3^ The advanced extended model of this mechanism emphasizes that the final equilibrium shift between protein conformations is the thermodynamic outcome of a multiscale protein-encoded dynamics that involves different length scales ranging from equilibrium atomic fluctuations to subdomain dynamics (flexible hinge regions or independent dynamic segments), up to large collective multidomain motions.^4−8^ Dynamic transitions are fundamental to trigger functional changes, and they can be seen as the protein response to a ligand, which acts as an external perturbation on a given conformational state. Dynamic changes explain how the receptor deals with this perturbation and how the latter propagates throughout the whole structure to stabilize the protein state that best adapts to the ligand.^7,9^ Since these states pre-exist in the native ensemble even in absence of any perturbation,^10^ they are likely to be intrinsically linked to functional modulation. Even when structural transitions are only subtle or not readily observed, the change in conformational landscape can still be linked to a population shift that involves energy redistributions or changes in the amplitude of atomic fluctuations.^9−11^

These considerations underpin the principles of orthosteric and allosteric functional modulation in all proteins and underlie affinity/efficacy discrepancies in docking-driven selections of the best compounds. Ligand docking strategies rely on a speed–accuracy compromise to efficiently screen, filter, and rank hundred thousands of ligands in a feasible time. In this framework, scoring functions prioritize ligands mainly approximating the enthalpic contribution due to protein–ligand interactions, while molecular dynamics (MD) has become a powerful instrument to take into account structure–dynamics–function relationships.^12−15^ In principle, MD offers atomic details of both the enthalpic and entropic contributions to the global binding free energy, possibly highlighting affinity and efficacy discriminants. In practice, this comes at the cost of performance, since a complete shift in the conformational equilibrium occurs on prohibitive time scales (microseconds to minutes). Nonetheless, the recognized potential of MD in the field is pushing significant efforts into extracting and analyzing MD trajectories of protein–ligand complexes at all levels, to inform *in silico* drug design and improve understanding of dynamic and functional differences resulting from ligand–protein cross-talks.^12−17^

Machine learning (ML) algorithms are being explored for trajectory data mining and with the purpose of extracting relevant information from MD trajectories collected for diverse bound/unbound conditions. In a series of recent papers, supervised and unsupervised ML techniques have been used as a comparative analysis tool for MD trajectories to classify and predict differential functional effects observed on GPCRs,^18,19^ PZD3 domain,^20^ and caspase-8,^21^ as a consequence of ligand binding. A software package (DROIDS 3.0) for the comparatively framed ML analysis of fast MD trajectories has been released to analyze the link between atomic fluctuations and functionally relevant protein regions affected by ligand binding or mutations.^22^ By transforming time-dependent Cartesian coordinates into ML-readable inputs, as images or matrices, ML algorithms demonstrated an ability to learn (training step) from known patterns (supervised ML) or find hidden ones (unsupervised ML) and are able to discriminate bound from unbound states. The sought-after patterns are intended as a particular combination of MD descriptors, also called features, which allow statistical classification of unknown trajectory points as belonging to one of the possible states (classes), e.g., inhibited versus activated states, on which the algorithm has been trained. Once an ML model has been internally trained and cross-validated for predictive performance, a step of external validation evaluates whether the learned patterns of descriptors are robust enough to generalize to a functional class from previously “unseen” trajectories. In these studies, a proper choice of comparatively framed MD-derived features over different time scales permitted a correct biologically and biophysically interpretation of MD-trajectories. Indeed, the sequential and extended conformational selection mechanism for binding stems from the key concept of hierarchy of time scales in proteins.^6,23^ Under this perspective, local ps–ns dynamic events can play a synergistic role in triggering slower transition by lowering energy barriers or increasing receptor’s probability to visit different states/substates.

Here, we made use of this principle to investigate the existence of ML-readable local dynamic patterns possibly connected to ligand-induced allosteric mechanisms that modulate the ATPase function in the molecular chaperone TRAP1. This mitochondrial member of Hsp90 family is a multidomain asymmetric dimer,^24^ where sequential ATP binding and hydrolysis in the two N-terminal domains (NTD) trigger allosteric conformational changes involving the large middle (LMD), the small middle (SMD), and the C-terminal (CTD) domains, more than 40 Å distal from the NTD.^25^ Allosteric inhibition of this target is now appreciated as an attractive strategy to selectively perturb TRAP1-dependent mechanisms involved in tumor growth, without interfering with other constitutive Hsp90 members.^26^

**Chart 1:**
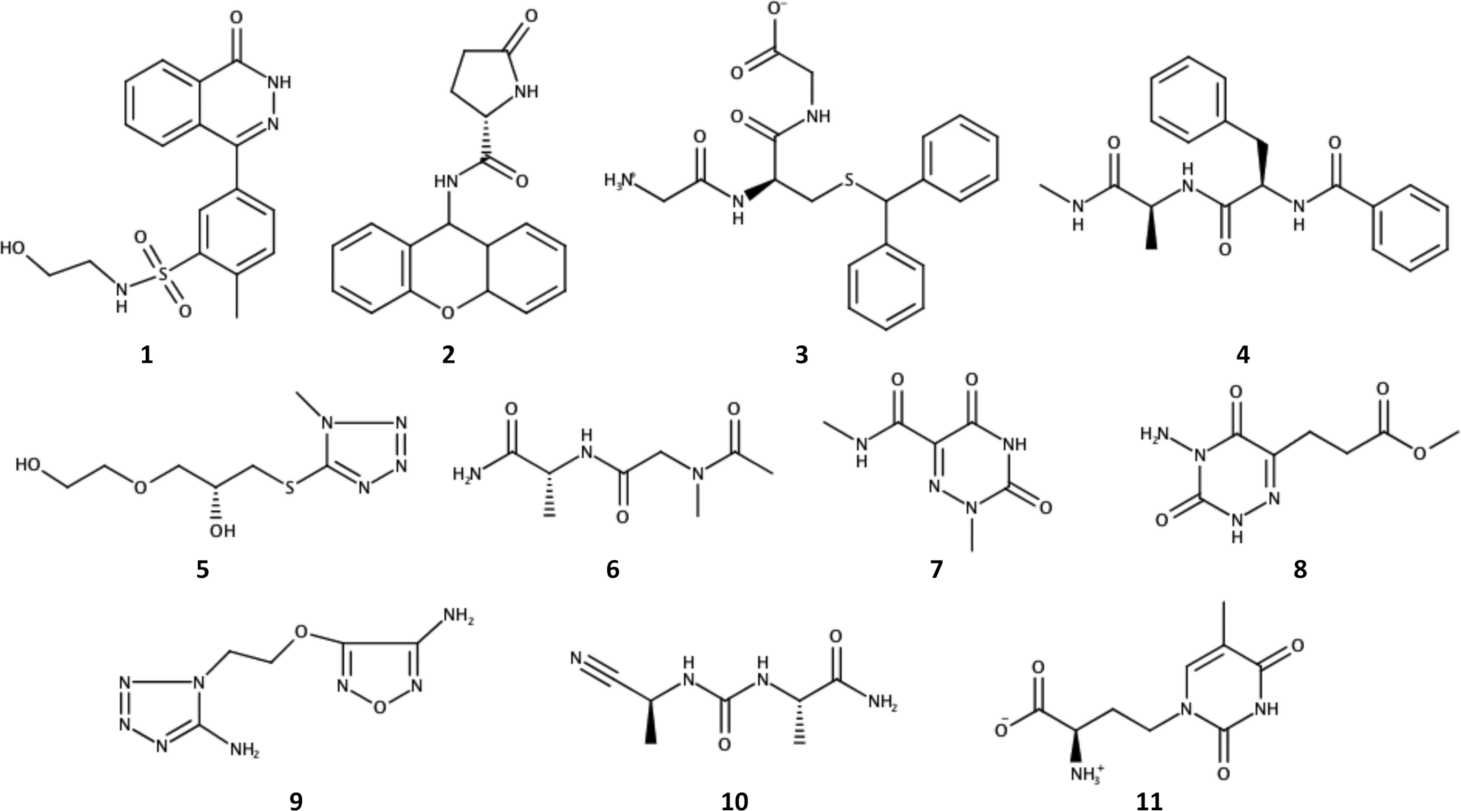
Chemical Structures of the 11 TRAP1 Allosteric Modulators Investigated in This Study

In this work, we tested the ability of ML to classify 11 novel allosteric modulators according to their effect on TRAP1 ATPase activity (Chart 1).

The experimental data in our hands set the stage for retrospective validation of the inhibitory efficacy of our compounds, which regardless of similar affinities to the target, decreased the chaperone ATPase function between 26.3 and 76%.^27^

Since the time scales required to fully explore end-point allosteric effects limit the use of MD for comparison of many bound/unbound states, an ML-based description of MD data was chosen in the attempt to rationalize compounds’ functional effects by focusing on the (apparently) noisy nanosecond dynamics of highly flexible subdomains with a well-established role in TRAP1 dynamics and function. A total of 66 MD systems were used to investigate the outcome of naïve Bayesian (NB) and support vector machines (SVM) algorithms. An ML-driven comparative-perturbed-ensemble analysis revealed a redistribution of inhibitor-bound/-free states observed as a consequence of ligand perturbation of a single low-energy TRAP1 conformation. Linear regression models were built to assess the relationship between the percentage of ML-predicted inhibitor-bound states and percentage of experimental TRAP1 inhibition. NB predictions returned regression models with maximum *r*^2^ between 0.64 and 0.71. This comparatively framed method for simulation and analysis provided a suitable ground to infer ligand functional effects within a coherently generated MD ensemble,^28^ enabling proper exploration of the potential of ML techniques applied to a challenging real-world case study.^29^ We show that a rigorous statistical ML framework can empower interpretation of different MD features in a perturbed ensemble, generating new knowledge to assist chemical biology and docking studies.

## Methods

### Generation of the Comparative MD Ensemble

The closed dimeric form of zebrafish zTRAP1 in its activated double ATP-bound state (Protein Data Bank ID: 4IPE)^24^ was simulated in inhibitor-unbound (state A) and inhibitor-bound (state I) to generate a ligand-perturbed conformational MD ensemble, whereby local functional TRAP1 dynamics could be compared in the presence and absence of the 11 allosteric inhibitors (Chart 1). All the compounds were docked in the same representative starting structure obtained via cluster analysis on a previous set of 600 ns MD simulations. We refer the reader to our original publication for technical details on the clustering procedure followed to extract the common initial TRAP1 configuration.^30^ This choice was done to select an equilibrated dimer conformation in a relaxed local minimum around the native crystallographic state. The backbone RMSD (1266 residues) between the chosen reference structure and the crystallized dimer was 3.83 Å. To enhance the sampling around the near-native conformation, each inhibitor-bound complex was independently simulated in 3 replicates, to get 33 ligand-perturbed systems. For comparison, 33 independent copies of the unperturbed TRAP1 system were simulated in the same conditions with only two ATP molecules bound to the two catalytically competent sites in the NTDs. Nine out of 33 inhibitor-bound systems and 3 out of 33 inhibitor-free replicates were taken from our previous MD simulations, in which 3 replicates for each of the 3 most active compounds (namely, compounds **5**–**7** in Chart 1) were compared with 3 inhibitor-free copies of TRAP1.^27^ Here, 24 inhibitor-bound systems and 30 inhibitor-unbound complexes were added to this initial set to complete the MD ensemble. The 3 replicates for each of the 8 discovered hits (namely, compounds **1**–**4** and **8**–**11** in Chart 1), as well as the new copies of the inhibitor-free state, were built following the same protocol used for the old simulations and described in full details in the original publication.^27^ Briefly, the Schrödinger software suite release 2017–1 was used for system setup.^31^ Flexible ligand docking into TRAP1 allosteric site was performed using Glide with default settings in SP mode.^32^ The best-ranking docked complexes were solvated by building a isometric truncated octahedral simulation box, leaving 10 Å solvent buffer from the protein. Equilibration and production steps were performed using the AMBER16 MD engine,^33^ describing the protein with the ff99SB force field and employing GAFF parametrization for ligands. Each independent complex, containing ≈175 000 atoms, was minimized and gently heated to 300 K, allowing volume and density equilibration in the *NPT* ensemble before switching to the *NVT* production run. In every replicate, velocities were reinitialized according to Maxwell–Boltzmann distribution at 300 K; the first 20 ns of each *NVT* run were discarded to allow for further system relaxation. Productive statistics was accumulated for a minimum of 80 ns to a maximum of 280 ns for every MD run, depending on whether the replicate was part of the training or the test sets used for ML analysis (see below). For the 66 systems, a total of 8.88 μs of production MD were collected and analyzed to build up features matrices of local MD descriptors. ML Classification tasks were later assessed by training and testing NB and SVM algorithms on combined matrixes obtained from each replicate.

### Generation of MD Features Matrices for ML Analysis

Four local features for each TRAP1 monomer were chosen based on theoretical and experimental evidence attributing to these subdomains structural and dynamic properties connected to the ATPase functional cycle. The rationale behind their choice is discussed in the “Results” section. The eight variables monitored along each MD trajectory were tested in a ML framework as “local reporters” of TRAP1 allosteric perturbation. Each MD frame was transformed in a feature vector of 8 TRAP1 descriptors (4 × monomer). The resulting features matrices contained the number of MD frames as rows and the 8 MD-derived features as columns. Descriptors were generated with in-house scripts using VMD (version 1.9.3)^34^ to calculate solvent accessible surface area (SASA) and *g_mindist* tool of gromacs (version 4.6)^35^ to get residue–residue contacts. Formal definition of the four MD descriptors are reported as follows:

Two sets of cross-monomer contacts were calculated between either NTD-swapped N-terminal extension (residues 85–108) in one monomer and the NTD core of the other (residues 109–308). In every frame, the number of contacts was obtained by summing up every pair of heavy atoms belonging to different domains, whose distance was lower than 4.5 Å.

Two sets of SASA values were collected for the ATP lid of both monomers in the NTDs of TRAP1. For the analysis, a rolling sphere of radius 1.4 Å was used to identify water accessible surfaces. Calculations were restricted to the side-chains of residues 191–217.

R417-γPO4 distances in the buckled and straight monomers were calculated between centers of masses of the gamma-phosphate group of ATP and the guanidinium group of R417.

Solvent exposure of S582 at the end of each of the two SMD–CTD linkers was calculated from SASA values (as above) of its phosphorylatable hydroxymethyl side-chain.

### Generation of Training, Validation, and External Test Sets from the Comparative MD Ensemble

Initially, 18 matrices, each built on 80 ns MD run, were merged in the so-defined “original” training set (Table 1) to represent 9 copies of the TRAP1 active state dimer (double-ATP-bound) in absence of inhibitors and 9 systems representing the same active state after binding of compounds **5**–**7** at the allosteric site. Every features vector belonging to these two groups was accordingly labeled as “A” (inhibitor-free) and “I” (inhibitor-bound) in the combined matrix. Since MD frames were saved every 20 ps, 80 ns of productive statistics returned individual features matrices of 4000 records. In total, the original training set contained 72 000 (4000 records × 18) data points describing 1.44 μs of aggregated statistics; every inhibitor-bound complex in the training set was evolved in MD for a cumulative time of 240 ns (80 ns × 3 repeats) and compared with an equal amount of statistics collected on the unperturbed TRAP1 state simulated in identical conditions.

**Table 1:**
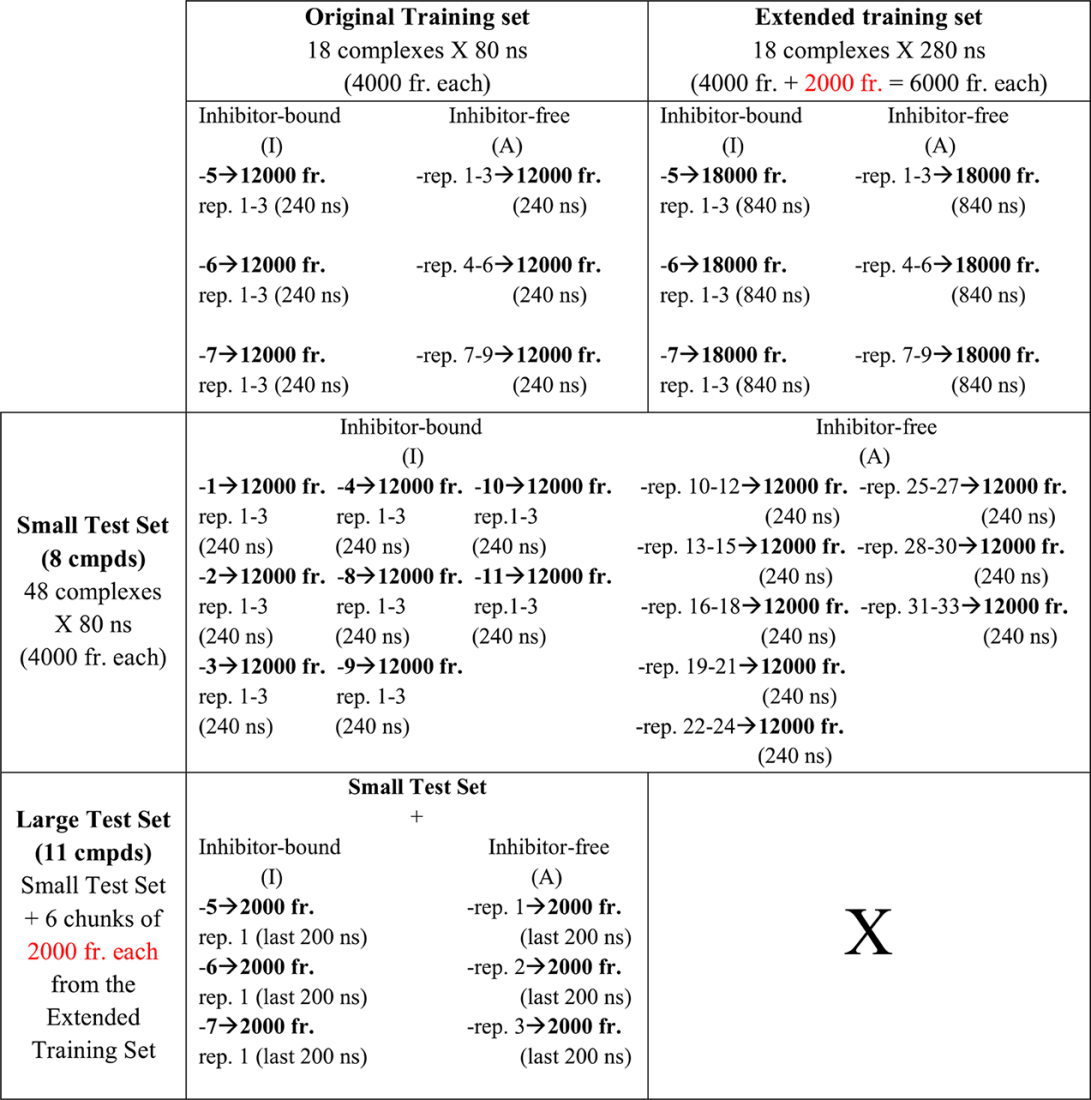
Training and External Test Sets Used for Comparative ML Analyses^a^

a Details on trajectories included in the training/test sets are reported in the corresponding cells. The small test set was used for external validation of both the training sets (merged row); extended trajectories of compounds (cmpds) **5**–**7** (red text) were “unseen” only by the original training set, so the extended training set was not validated against the large test set (X).

The predictive power of the trained models was internally checked via 5-fold cross validation procedure available in MATLAB version 2019b,^36^ by randomly using 20% (14 400 records) of the training set as validation set in each fold (see below). To test the effects of increased sampling on the predictive power of our MD descriptors, a so-defined “extended” training set was generated by retraining the algorithms after the addition of 200 ns to each of the 18 systems belonging to the original training set (Table 1). To keep the size of the data set reasonable, a features vector was built every 100 ps, and 2000 new records were added to each original matrix. The extended training set included a total of 108 000 features vectors collected over 5.04 μs of aggregated statistics; every replicate of the three inhibitor-bound complexes was extended to reach a cumulative time of 840 ns (280 ns × 3 repeats) and similarly compared with an equal amount of statistics collected on the unperturbed TRAP1 state simulated in identical conditions. A 5-fold cross validation was used for internal accuracies as described for the original training set.

External performances of the original and extended training sets were verified against a so-defined “small” test set including the less active compounds of the library (compounds **1**–**4** and **8**–**11** in Chart 1), each consistently simulated over 3 independent replicates for 80 ns. A total of 24 TRAP1 complexes (8 ligands × 3 repeats) was used to build a features matrix of 96 000 data points (4000 records × 24). The small test set was properly balanced by the addition of an equivalent number of 96 000 data points collected from 24 replicates of the inhibitor-free state and simulated in the same conditions. A total of 192 000 unlabeled and unseen features vectors were subjected to ML predictions based on 3.84 μs of aggregated statistics.

In order to test models external performance against compounds **5**–**7**, the final ML models trained on the original data set were used to predict TRAP1 states from 200 ns long trajectories, which had been used to build the extended training set (Table 1). Indeed, the extended portions of these trajectories were not part of the original training set and were treated as out-of-model data for this latter. Three features matrices, each of 2000 unlabeled records (time interval: 100–300 ns) from the extended training set, were thus added to the small test set (8 ligands). The so defined “large test set” was then used to make predictions on 11 inhibitor-bound systems. The large test set was properly balanced by adding three more matrices of 2000 records each, similarly extracted from the extended portions of three inhibitor-free trajectories. The large test set contained only one replicate of compounds **5**–**7** to ensure similar statistics and time scales were used for prediction of all ligand-bound states (200 ns for compounds **5**–**7** and 240 ns for the others). A set of 12 000 additional data points (1.2 μs) were added to the small test set, totaling 204 000 “unseen” records of featurized MD frames (see Table 1).

### Comparative Performance Analysis of ML Classification Tasks: Naïve Bayesian Algorithm and Radial Basis Function Support Vector Machines

Two supervised ML algorithms based on generative and discriminative approaches were used to explore models ability to predict meaningful local patterns representative of bound (I) and unbound (A) TRAP1 states. Two variants of the NB classifier were chosen to exemplify the performance of generative models, while SVM based on Gaussian radial distribution functions (GDF-SVM) was chosen as representative of discriminative models. The main difference between generative and discriminative algorithms resides in the way they make decisions to separate I from A states in the defined eight-dimensional features space.

NB classifiers are probabilistic algorithms that generate statistical distribution models for the classes and for individual features within the classes, estimating prior and posterior probabilities from empirical evidence in the training set according to Bayes’s theorem and considering features independence (naïve assumption). Depending on the shape of data, Gaussian (GNB) or continuous (KNB) kernel functions are used to calculate joint probabilities for features and classes (for methods, see the Supporting Information).

In contrast, the radial basis function SVM consists of discriminative nonprobabilistic algorithms which learn the best decision boundary between classes given an input *n*-dimensional space. When data are not linearly separable, SVM transforms data through kernel functions that map from each point in the input eight-dimensional space to the corresponding class in the so-called kernel space. This mapping strategy enables to find a linear boundary between classes in this new space. Gaussian distribution functions are used in this SVM implementation as similarity functions to distinguish close from far features vectors in the mapped space (see the Supporting Information). SVM find the best separating hyperplane based on the sampling of the input space. In other words, SVM does not address the probability that connects members of a class to the ensemble of visited features. Unlike NB, discriminative approaches do not take into account the statistical distribution of the features in the examined classes, but rather focus on the final distribution of actually visited coordinates in the space of the features. The reader is also referred to excellent reviews for further details.^37,38^

### ML Parameters and Performance Metrics for Internal and External Validations

GNB, KNB and GDF-SVM were trained on the original and extended training sets (Table 1) by using the Classification Learner app available in the Statistics and Machine Learning Toolbox of MATLAB.^36^

To minimize overfitting, the models were subjected to 5-fold cross-validation during training. The data set was randomly splitted in 5 subsets of roughly equal size in a 80:20 ratio. In five iterations, each model was trained on the 4 subsets and subsequently validated on the remaining one to ensure each subset was considered as a validation set at least once. Then, the overall performance of the models was assessed by comparing cross-validation accuracy values, reported as the average of single accuracies (*Q*) obtained in each fold described as
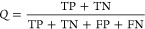
1In our ML models, states classified as I were treated as true positives (TP), while states classified as A were treated as true negatives (TN). Here, false positives (FP) represented frames classified as I in inhibitor-free trajectories, while false negatives (FN) were the frames classified as A in inhibitor-bound systems.

NB was trained using Gaussian kernel coupled with parametric (GNB) or nonparametric (KNB) distribution functions available in MATLAB. Both the NB algorithms were externally validated on the two out-of-model test sets. GDF-SVM was trained tuning the hyperparameters σ and *C*, i.e., *kernel scale* and *box constraint* values, respectively. MATLAB uses an heuristic methodology to train three presets referred to as coarse, medium and fine, where *C* was fixed to 1 and the kernel scale was set at 11, 2.8, and 0.71, respectively.^36^ Moreover, a Bayesian optimization procedure was carried out to perform a statistics-based evaluation of σ and *C* values. Bayesian optimization does an informed and efficient search in the space of hyperparameters based on a probabilistic model and an iterative approach that minimizes the score of the classification error function. The σ and *C* hyperparameters were tuned in the range 0.001–1000. Two rounds of optimization were performed on the two training set, using 15 and 40 iterations. The software selected the best models as those that minimize the upper confidence interval of the classification error function. To minimize the risk of overfitting, the 5 GDF-SVM models (3 presets and 2 optimized) trained on the original and extended training set were externally validated on the small and large test sets. Only the two GDF-SVM models (one for the original and one for the extended training set) with the best external performance were selected for further investigation.

For all NB and GDF-SVM models, true positive (TPR) and true negative (TNR) rates were used as specific performance metrics to express training and test set accuracies for states I (TP) and A (TN), respectively. TPR an TNR values consider the number of predicted positives and negatives over the total number of true positives and negatives in the data set and can be calculated as follows:
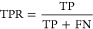
2
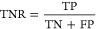
3False positive rates (FPR) can be directly calculated from TPR as follows
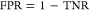
4The predicted percentages of TP and FP from external inhibitor-bound and inhibitor-free trajectories, respectively, were plotted against the corresponding percentage of TRAP1 inhibition calculated from experimental assays. Linear regression models were generated on the predicted vs observed variables with the Curve Fitting tool of MATLAB.^36^ The coefficient of determination (*r*^2^) was reported to measure the percentage of variability in the experimental data that can be expressed by the variance resulting from ML predictions.

## Results

### Theoretical and Experimental Background for Features Selection and Data Partitioning

**Figure 1:**
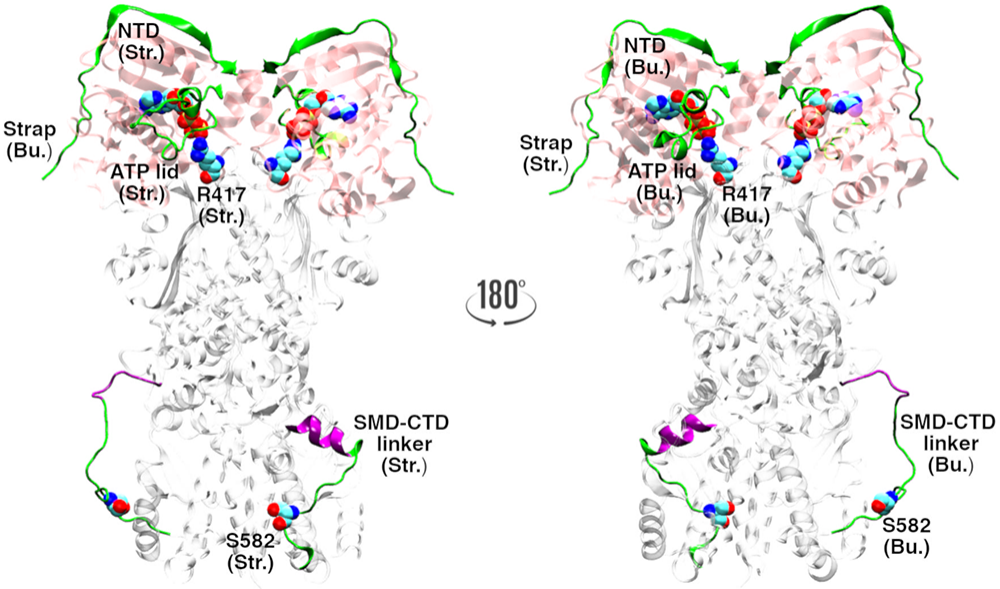
MD descriptors mapped onto 180°-rotated views of the buckled (Bu.) and the straight (Str.) TRAP1 monomers in the active asymmetric state. Two ATP molecules are bound to their pockets in the NTDs (pink) and establish the featuring salt-bridge with R417 on the ATP sensor loop (vdW spheres). Protein segments with enhanced local dynamics within the dimer are shown (green) and labeled accordingly. The two NTDs make cross-monomer interactions with the N-terminal strap of the partner monomer. S582 (vdW spheres) is shown in the SMD–CTD linker; the segment 566–572 (purple) is highlighted in its ordered (straight) and disordered (buckled) structure. For clarity, in each view, labels are reported for the front monomer only.

Rational selection of TRAP1MD descriptors was carried out restricting our choices to dimer subdomains with demonstrated key roles (simulations and experiments) in TRAP1 conformational dynamics and ATPase function. We focused on residue-level solvation, contacts, and distances obtained from four inherently flexible regions of the buckled and straight monomers, belonging to NTD, SMD–CTD linker, and the ATP sensor loop in the LMD (Figure 1).

On the basis of our previous findings,^27^ the allosteric perturbation induced by compounds **5**–**7** strongly affected the global NTD flexibility, including NTD buckling motions in the catalytically competent monomer. The ATP binding pocket in the NTD, which displayed efficient long-range communication propensity with the allosteric site,^27,30^ contains two higly flexible elements which are well-known kinetic regulators of TRAP1 conformational changes along the ATPase cycle: the N-terminal extension and the ATP active site lid.

These two regions are responsible for functional cis-/cross-talking within and between the two NTDs and directly respond to nucleotide binding to mediate dimerization, to induce the “tense” active state or to relax the dimer in a set of open apo forms.^39,40^

The N-terminal extension, also called the “strap” (segment 85–100), behaves as a large thermal barrier to closing and opening motions by stabilizing interactions with the *trans*- and the *cis*-NTD, respectively.^41^ Mutants or strap-truncated TRAP1 constructs resulted in a dramatic increase in ATPase activity from 3- to 6-fold in zTRAP1 and even to a 30-fold increase for hTRAP1.^24,41^ Our recent MD study has shown that the fragment undergoes abrupt changes in internal stiffness and mechanical coupling with both the NTDs when the most effective compounds were simulated in the allosteric site for 900 ns.^27^

The ATP lid (residues 191–217) is essential for dimerization and the ATP hydrolysis reaction.^40,42^ Local and global motions between open and closed conformations are affected by ligand binding;^43^ in fact, previous simulations on the cytosolic Hsp90 showed that in the double ATP-bound state the ATP lid is widely flexible.^44^ A similar behavior has been experimentally observed also in TRAP1,^24,41^ suggesting that local dynamics in this region is not suppressed by nucleotide binding.

Even if motions at the level of these two TRAP1 motifs represent some of the rate-limiting steps needed to promote slower conformational changes, experiments reported that the two structural elements enjoy differential plasticity on faster time scales and sense the chaperone binding state to trigger oriented and functional conformational transitions.^40^ Here, we monitored the effects of the allosteric perturbation on their plasticity, assessing whether ligand-induced strain at a distal site could propagate to the NTD and perturb the local dynamics of long-range communicating regions in a meaningful way. Such an effect was sought in changes in the ATP lid SASA and cross-monomer contacts involving the strap motifs and their partner NTDs.

The third local feature we investigated was the interaction distance between the catalytic R417 and the ATP gamma-phosphate group. The arginine resides in the LMD on the ATP-sensor loop and readily responds to the nucleotide to trigger loop rearrangements required for catalysis.^45^ The interaction is the fingerprint of the catalytically competent ATP-bound state and differentiates the internal dynamics of the active state from ADP-bound or apo forms, where this salt bridge is missing.^25,30^ Moreover, since this amino acid was critically involved in the establishment of long-range coordination with the allosteric site, we expected that a greater responsiveness to allosteric perturbation could reverberate on the stability of this functional ion pair.

As a fourth variable, we focused on the solvent-accessibility properties of S582 located in the structurally disordered TRAP1 SMD–CTD linker (residues 572–586). Mutagenesis studies by Masgras and co-workers identified this serine residue as an accessible phosphorilation site for ERK1/2 on the closed dimer, which enhances TRAP1 chaperone activity.^46^ In several tumor models, TRAP1 down-regulates succinate dehydrogenase (SDH),^47^ leading to transcriptional changes that ultimately favor the advantageous metabolic switch from OXPHOS to aerobic glycolysis in aggressive neoplasms.^48^ Since our tested allosteric inhibitors result in a beneficial increase of SDH activity *in vitro* and *in vivo* tests,^27^ we monitored differences in S582 solvent exposure in the two simulated states as a consequence of allosterically perturbed dynamics. Notably, the helix preceding the SMD–CTD linker in the crystal structure, that is helix 20, contains a residue (E566) which makes asymmetric interactions in the straight protomer and if mutated decreases ATPase activity of more than 60%.^24^ In contrast, the same helix is an unstructured fragment in the buckled monomer, including 20 highly flexible and disordered residues (566–586). This aspect is reminiscent of intrinsically disordered regions in folded complexes, which are arising much interest because of their role in allosteric regulation of multidomain proteins.^49,50^ These regions can locally fold or unfold to dissipate sources of mechanical stress in the dimeric asset and thus trigger sequence-encoded dynamics changes eventually connected to complete state transitions.^10,51,52^ Notably, the whole N-terminal region (residues 85–108) is composed by the “strap” extension (residues 85–100) and the beta-strand (residues 101–108) in the closed dimer. However, the two subregions are α-helical in a recently crystallized apo form of the hTRAP NTD construct.^39^ Structural differences in structural order are also observed for the ATP lid in the two crystallized zTRAP1 monomers: While the buckled monomer displays a helix–loop–helix fold, a portion of the ATP lid is missing in the straight protomer and the helical regions are partially unfolded.^24^ These considerations suggest that changes in folding could involve key regulatory elements of the chaperone.

Given the above-described set of features, three aspects were taken into account in the generation of the training sets: (i) The 11 ligands inhibit TRAP1 function in a relatively wide range of activities, and such an heterogeneous data set could in principle contain spurious inhibited states (I), which might poorly represent the effects of the most active compounds. (ii) The emergence of local dynamics patterns was searched as a result of the allosteric perturbation in a unique initial structure, so we needed to maximize the diversity between substates accessible to inhibitor-bound and inhibitor-free systems. (iii) Since short-time-scale MD on a single replicate is severely affected by the sampling problem, more replicates of a single complex were simulated to improve data statistics.

In the attempt to tackle these specific issues, only the three most effective inhibitors (compounds **5**–**7**) with almost homogeneous inhibitory efficacy (73–76%) were used in the training set and balanced with the same number of inhibitor-free replicates. In other words, ML models were built to ensure learning of inhibitor-bound patterns from the allosteric ligands expected to have the biggest detectable impact on TRAP1 local unperturbed dynamics.

### Probability Distribution Plots of Local Features in A and I States

**Figure 2:**
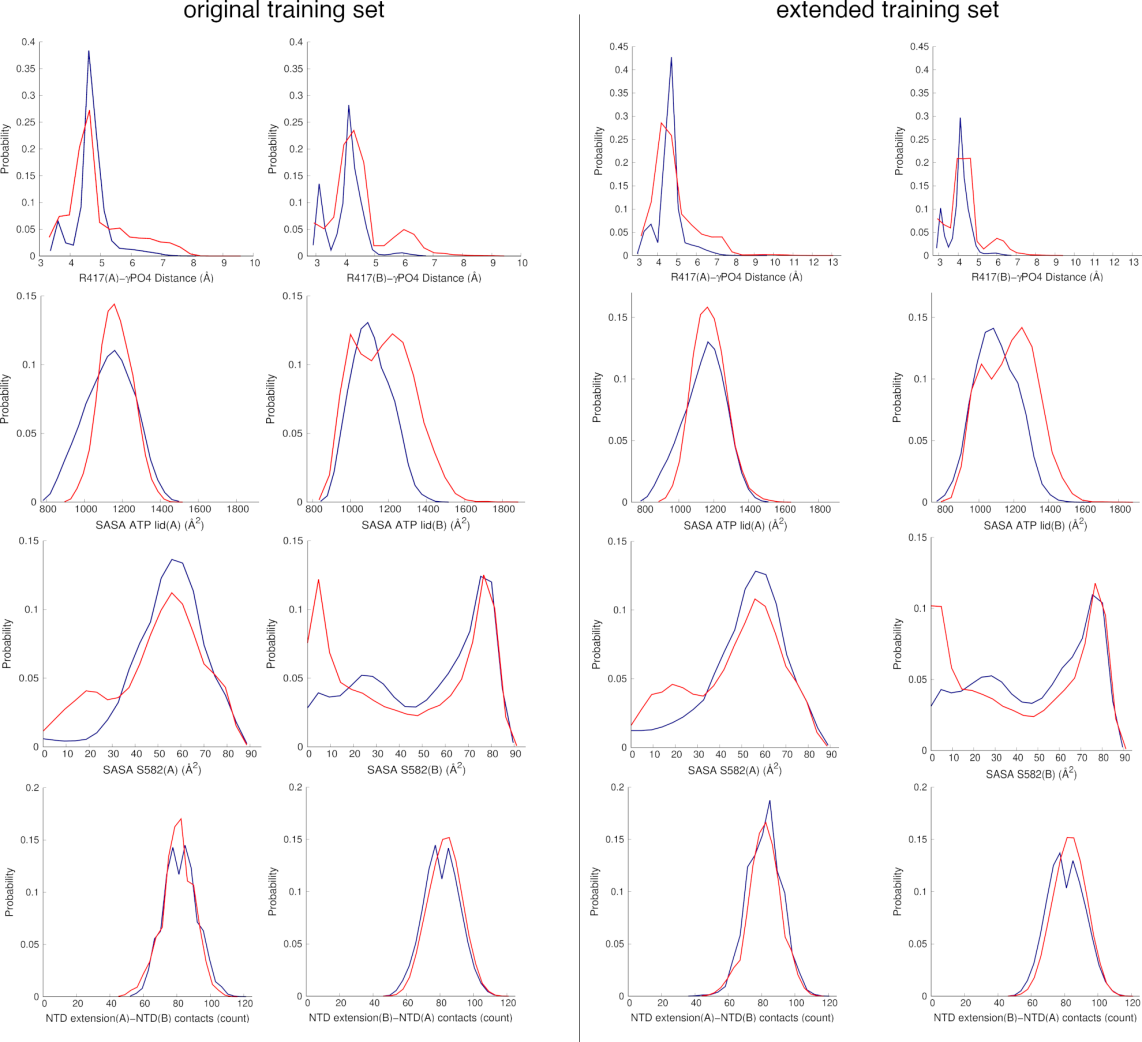
Probability distributions for the eight features in TRAP1 states A (blue) and I (red) in original (720 ns for each state A/I) and extended training sets (2.52 μs for each state A/I). The plots were obtained distributing individual features vectors collected from 9 inhibitor-unbound replicates and 9 inhibitor-bound complexes containing compounds (**5**–**7**) with the highest inhibitory efficacy in the allosteric site (see Table 1).

Figure 2 shows the degree of sampling achieved by each variable in the 9 inhibitor-free (state A) and 9 inhibitor-bound (state I) complexes from the original and extended training sets (Table 1). Since states A and I were simulated starting from the same reference TRAP1 structure, it is not surprising to notice overlap between the visited space on the simulation time scale. Although states I are also accessible to states A to a certain extent, the three most active compounds of the series (**5**–**7**) cause sensitive and heterogeneous perturbations of the statistics sampled by inhibitor-free systems, affecting the modality of the distributions and the ensemble probability of the different peaks in the features space. Both the training sets highlight interesting ligand-induced differences within a few features distributions, which cannot be observed in the test set (see Figure S1), as in the case of the SASA values for S582 and the ATP lid (Figure 2). The addition of 200 ns of statistics in the extended training set do not substantially change the shape of distributions for state A. Furthermore, the 9 inhibitor-free systems in the training set and the 24 inhibitor-free replicates included in the test set appear to visit overlapping regions of the features space (see Figures 2 and S1). Nevertheless, the curves clearly show poor separability of TRAP1 states, demonstrating that the classification task cannot be based on a simple analysis of the probability plot nor can differences between the states be rationalized. These considerations set the stage to turn to ML classification tasks, in the attempt to expose emergent local and functional dynamic patterns characterizing the MD ensemble.

### Internal Performance of NB and SVM Models on Validation Sets

Specificity (TNR) is considered as a more robust metric than sensitivity (TPR) to validate our models. It can be stated that if states labeled as A (TN) in the training sets are genuinely meaningful of an unperturbed local equilibrium learned from TRAP1 inhibitor-free dynamics, external trajectories of the same type should reproduce this behavior.^22^ Furthermore, a specific model is desirable in a supportive tool for drug design, since it should minimize prioritization of inactive compounds (FP). Specificity is generally lower but should not drastically degrade in external testing. In order to build ML models as robust as possible, we relied on their ability to predict states A, learnt from trajectories of 36 000 or 54 000 frames, in a much larger number of out-of-model inhibitor-free trajectories available as external test set (96 000 frames). Sensitivity (TPR) could depend more on the extent of perturbation induced by different allosteric modulators, so a lower sensitivity may also be descriptive of ligand functional properties and could not necessarily imply bad prediction accuracy for states I. However, as a compromise, we excluded the GDF-SVM models with the lowest TPR and selected the one with the highest TNR among the other four SVM setup. Table 2 summarizes , TPR, and TNR metrics calculated via 5-fold cross-validation of GNB, KNB, and GDF-SVM algorithms. For the latter, the “medium” preset (σ = 2.8 and *C* = 1) returned the best performance in the external validation procedure and was selected for further comparative analyses. Interestingly, the overall prediction accuracy slightly decreased when the simulations were extended up to 300 ns, indicating that sampling in the first 80 ns (in each replicate) produced more identifiable patterns of perturbed (TPR) and unperturbed (TNR) complexes.

**Table 2:** Internal Cross-Validated Performances of Generative and Discriminative ML Models^a^

	GNB			KNB			GDF-SVM		
	⟨*Q*⟩%	TPR	TNR	⟨*Q*⟩%	TPR	TNR	⟨*Q*⟩%	TPR	TNR
original training set	73	0.63	0.83	78.9	0.77	0.81	94.9	0.95	0.95
extended training set	66.7	0.55	0.79	74	0.70	0.78	91.0	0.92	0.92

a Cross-validated percentage accuracy  and corresponding TPR and TNR for GNB, KNB, and GDF-SVM models (“medium” preset: σ = 2.8 and *C* = 1). Performance metrics were calculated summing up TP and TN predictions on the five validation subsets generated from the training data.

Such a result indicates that although starting from the same reference structure, on the shortest simulated time scales, the chosen local features experience more relevant perturbations relative to TRAP1 inhibitor-unbound states that come partially restored or become less identifiable extending the simulations by 2.5-fold.

The average accuracy in each validation fold varies between 66.7 and 94.9%, with the discriminative models outperforming the generative ones in the internal validation. All the models are characterized by good internal specificity (TNR), recognizing the A state in a range between 78 and 92% of the inhibitor-free trajectories. In contrast, model sensitivity (TPR) differs more among the models, identifying state I in a range between the 55 and 95% of the inhibitor-bound frames. Overall, GDF-SVM gives models of high sensitivity for state I and specificity for state A in the training set, whereas NB shows good specificity for state A, reaching 0.83 when data are modeled as Gaussian distributions (GNB) but lower ability to identify all inhibitor-bound frames as genuine states I. In KNB models, nonparametric statistical treatment of the data set returns the highest TPR values. In order to evaluate ML predictions over a fixed simulated time window and allow coherent comparison among homogeneous inhibitor-bound and inhibitor-free trajectories, Table 3 reports the percentages of correct predictions (TP and TN) in the two training sets for aggregated trajectories (12 000 or 18 000 frames) grouped for each simulated ligand (3 replicates). The internal performance metrics of our models remarks the absence of a clear separation between the sampled states in the ensemble; rather, states A and I seem to coexist as a minor population in inhibitor-bound and inhibitor-free trajectories, respectively. The trained models were able to recognize no less than 49% of states I in each individual inhibitor-bound state and no less than 69.5% of states A in inhibitor-free ones, highlighting their preference for one of the two, without excluding minor sampling of the other.

**Table 3:** Internal Validation Metrics Reported as TPR % and TNR % for Individual Systems in the Bound/Unbound States^a^

		original training set		extended training set	
ML models	TRAP1 complexes	TPR % (state I)	TNR % (state A)	TPR % (state I)	TNR % (state A)
GNB	5	71.4		49.8	
	7	63.0		52.0	
	6	61.2		62.1	
	inhibitor-free (rep. 1–3)		69.9		75.1
	inhibitor-free (rep.4–6)		84.5		80.3
	inhibitor-free (rep.7–9)		90.0		80.2
KNB	5	70.9		78.3	
	7	94.2		63.9	
	6	66.6		68.8	
	inhibitor-free (rep. 1–3)		70.1		69.5
	inhibitor-free (rep.4–6)		89.6		83.4
	inhibitor-free (rep.7–9)		82.9		78.4
GDF-SVM	5	92.5		90.9	
	7	97.3		93.7	
	6	95.4		91.3	
	inhibitor-free (rep. 1–3)		95.7		92.7
	inhibitor-free (rep.4–6)		98.4		96.7
	inhibitor-free (rep.7–9)		92.5		89.7

a Percentages are shown over chunks of 12 000 (original training set) or 18 000 (extended training set) frames. Models trained on the entire training set were used to make predictions.

### External Performances of the Models on out-of-Model Test Sets

External testing of the models causes an expected decrease in TPR and TNR, relative to the validation sets (Table 4). In spite of the 24–27% loss in the specificity of SVM models, 68–69% of inhibitor-free trajectories can be genuinely recovered as TN. For NB models, the loss in specificity is lower, in the range of 2–23%. Training NB models on the extended data set leads to a lower loss in specificity in respect of values obtained from the original training set. Specifically, the GNB model trained on the extended data set, almost retains the same specificity (0.77) observed during internal validation (0.79), whereas a higher but acceptable 13% loss in TNR is obtained with KNB. Adding sampling to the original training set improves recognition of states A on a much larger number of “unseen” inhibitor-free replicates. Overall, data modeling via normal distributions (GNB) provides the most specific models for state A predictions, while the use of nonparametric statistics in the KNB variant returns increased sensitivity toward the state I, although the overall TPR values are significantly lowered in the external test sets.

**Table 4:** Performance Metrics for the External Validation of the Three ML Algorithms Trained on the Original and Extended Dataset^a^

		GNB			KNB			GDF-SVM		
training set	test set	TNR	TPR	*r*^2^	TNR	TPR	*r*^2^	TNR	TPR	*r*^2^
original	small	0.68	0.38	0.50	0.59	0.42	0.45	0.69	0.40	0.11
	large	0.67	0.41	0.64	0.58	0.44	0.61	0.68	0.42	0.53
extended	small	0.77	0.31	0.56	0.65	0.37	0.71	0.68	0.37	0.18

a TNR (states A) and TPR (states I) are extracted from 96 000 and 102 000 MD frames for each state in the small and large test sets, respectively. The *r*^2^ values obtained from linear regression analyses are also shown for each set of predictions, with the best values highlighted in red.

**Figure 3:**
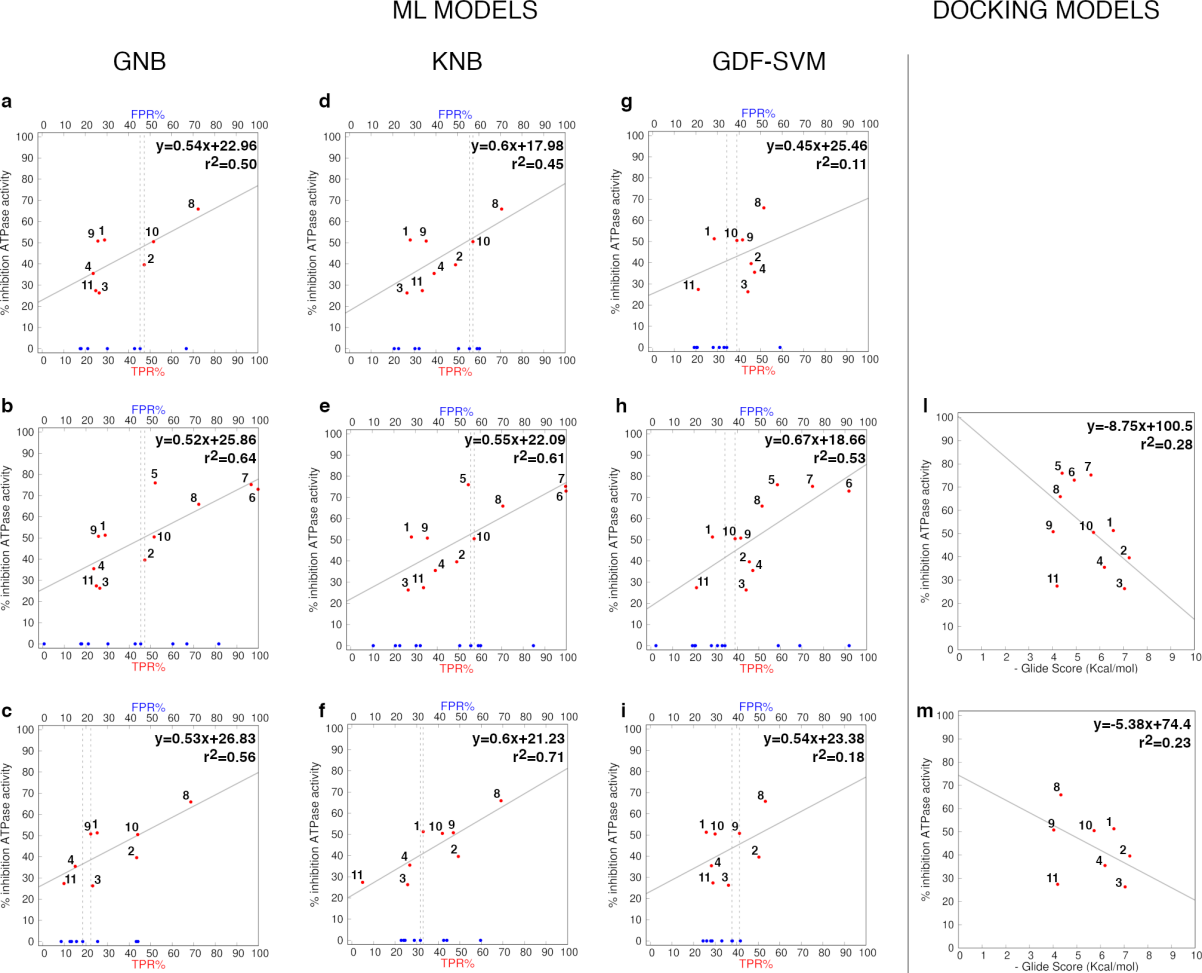
External validation of GNB, KNB and GDF-SVM models against small and large test sets. Predicted TPR percentage (TPR%, red dots) for the 8 ligands (a, c, d, f, g, i) or 11 ligands (b, e, h) against observed percentage of TRAP1 inhibition. In each plot, FPR percentage (FPR%) are calculated as the percentage of states I in the same number of inhibitor-free systems. ML models validated on the original training set (a, d, g) and the extended training set (c, f, i) were used for predictions. The original training set was also tested on “unseen” trajectories of compounds **5**–**7** (large test set) (b, e, h). Regression lines are shown in solid gray lines, with the associated equations and *r*^2^ values. Ligands are numbered as in Table 4. Dashed gray lines identify boundaries between A/I states: the first line from the left passes through the blue point that defines the maximum FPR% found in at least 62.5% of inhibitor-free trajectories; the second line from the left goes through the first TPR% point (red) found immediately after the first boundary and delimits a region where predicted states I in the inhibitor-bound trajectories (TPR%) is significantly greater than the threshold of states I characterizing the inhibitor-free trajectories (FPR%). Regression models built from docking scores on the small (l) and large (m) test set are shown for comparison.

ML predictions on the two external test sets are plotted on the *x*-axis of two-dimensional graphs using TPR percentage and FPR percentage values, describing the percentage of I states in inhibitor-bound and inhibitor-free trajectories, respectively (Figure 3a–i). Here, we calculate these performance metrics grouping TRAP1 replicates bound to the same ligand in every training/test set pairs. For comparison, results on inhibitor-free trajectories are similarly shown for groups of three replicates. Therefore, 8 or 11 predicted values are plotted against the experimentally observed percentage of functional ATPase inhibition (*y*-axis) (Table 5). FPR percentage predictions in inhibitor-free trajectories have *y* coordinates equal to 0 to represent lack of functional inhibition in absence of ligand perturbation. In order to assess whether ML predictions on inhibitor-bound systems can provide any meaningful correlation with observed functional inhibition, linear regression models are built on TPR percentage, to quantify the percentage of experimental variance explained by predicted values. Thus, the coefficients of determination (*r*^2^) for each model are presented as an additional metrics of external performance. Given the nonseparable nature of our data (Figure 2) and our choice to simulate near-minimum conformations sampled from a single reference structure, full segregation of states was not expected.

**Table 5:** Percentage Decrease in TRAP1 ATPase Function after Treatment with the 11 Allosteric Inhibitors Investigated in This Study^a^

inhibitor-bound TRAP1	% TRAP1 inhibition
**5**	76.0
**7**	75.2
**6**	73.0
**8**	65.9
**1**	51.3
**9**	50.8
**10**	50.5
**2**	39.6
**4**	35.5
**11**	27.4
**3**	26.3
inhibitor-free TRAPl	0.0

a Functional assays are described in our previous publication.^27^ Ligands are numbered as in Chart 1 and are ordered by decreasing effects.

Also, the functional heterogeneity of the tested inhibitors is not excluded to play a key role in limiting sampling of unambiguous I states. Nonetheless, the plots highlight interesting trends within and between predicted observables representing the A/I states. Direct comparison of I states in inhibitor-bound (TPR percentage) and inhibitor-free (FPR percentage) trajectories provided us with a consistent framework for internal assessment of TRAP1 patterns featuring its local internal dynamics, before and after the allosteric perturbation.

In each plot, we tried to identify two regions based on the segregation of A/I states along the *x*-axis (Figure 3a–i); two dashed gray lines parallel to the *y*-axis are drawn to identify boundaries in this two-dimensional space. The methodology is inspired by the principle used to identify support vectors in SVM algorithms.^53^

The first line encountered from the left passes through the maximum FPR% characterizing at least 5/8 (Figure 3a,c,d,f,g,i) or 8/11 predictions (Figure 3b,e,h) (62.5 and 72.7% of the observations, respectively) on inhibitor-free systems (blue dots). The second dashed line passes through the first TPR% value on the *x*-axis above that FPR% threshold. Depending on the model specificity, this geometric construct enables separation of inhibitor-bound/inhibitor-free systems, based on the percentage of states I (TP) predicted in each system of the ensemble. An inhibitor-bound state is assigned to class I if the TPR% in each of the 8 or 11 meta-trajectories is greater than the maximum percentage of false states I (FP) predicted in at least 5/8 or 8/11 inhibitor-free systems.

Comparing the external performance achieved on the small test set by GNB and KNB models trained on the original and extended training data (Figure 3a,c,d,f), we observe that the more specific models trained on the extended set shifts FPR% thresholds left by more than 20% (Figure 3c,f). In all the NB models tested on the small test set, the most active compound **8** increases the percentage of states I more than any other inhibitor-free system. However, if the NB models are trained on shorter MD simulations, then only two of the most active ligands, that is, compounds **8** and **10**, significantly shift the percentage of states I above the FPR% threshold predicted for the two models (Figure 3a,d). Compounds **1** and **9**, with inhibitory efficacies > 50%, as well as the less active compounds, do not segregate from inhibitor-free trajectories. Moreover, the regression models do not capture significant correlation (*r*^2^ > 0.60) between predicted TPR% and experimental inhibition data. Training NB on the extended set decreases the sensitivity of the models and increases specificity (Figure 3c,f), showing a significant *r*^2^ value of 0.71 for the KNB model. The plot in Figure 3f allows to classify states according to a linear model that is able to link ML predictions to functional differences among the ligands in a meaningful way. The region of the I state in this graph contains 5/8 inhibitor-bound trajectories, while 5/8 inhibitor-free states fell in the space mainly populated by the A state. Here, the remaining 3 ligands misclassified by the separating plane are actually the less active compounds with 26.3 and 35.5% inhibitory efficacy against TRAP1. However, if GNB models are used to classify the compounds, then the lack of significant correlation would lead to also prioritizing the worst compound of the series (ligand **3** in Figure 3c). As shown by the lower sensitivity and *r*^2^ values, the high specificity achieved in this model (0.77) is not balanced by a sufficient understanding of meaningful dynamic patterns.

The discriminative GDF-SVM models tested on the small series are highly specific and identify boundaries with larger margins between the states (Figure 3g,i). However, the lack of meaningful correlation between predicted and observed variables allows only a qualitative classification. 6/8 ligands are correctly found above the FPR% threshold characterizing 87.5% of the predictions made on inhibitor-unbound systems (Figure 3g). Even without showing meaningful correlation with experimental inhibition, SVM trained on the extended set provides higher sensitivity only toward the most active inhibitors, filtering out less active compounds **3** and **4** (Figure 3i). Indeed, if SVM models are trained on the extended data set, then only the three ligands (**2**, **8**, and **9**) with high to intermediate inhibitory efficacy fall above the TPR% boundary threshold.

The testing of ML models trained on the original set against “unseen” trajectories of compounds **5**–**7** increases the *r*^2^ values of all the corresponding regression models (Figure 3b,e,h), with NB models returning lower values of 0.64 (GNB) and 0.61 (KNB). Indeed, all the tested ML models are able to assign the highest relative TPR% to these three compounds. Even if the GNB model tested on the large set is less specific, then six ligands are associated to TPR% larger than the maximum FPR% threshold which characterizes the 72.7% of the predictions on inhibitor-free trajectories. As indicated by lower correlation (0.61) and by the lowest specificity (0.58), the GNB model outperforms KNB model on the large test set (Figure 3b), as the former is able to isolate the six allosteric modulators with the best pharmacological profiles from the region of the A states. Nonetheless, among the most active compounds, KNB correctly predicts four compounds (**6**–**8** and **10**) with TPR% above the maximum FPR% threshold (Figure 3e).

Concerning the performance of the discriminative SVM model on the large set, the significant increase in the *r*^2^ value is due to the capability of this model to assign the highest TPR% values to trajectories of compounds **5**–**7** (Figure 3h). Even if GDF-SVM models can identify ligand-perturbed states in a more qualitative manner, then the model shows the greatest sensitivity for the most active compounds, **5**–**7**. Here, we notice that states I for ligands **5**–**7** are predicted from the extended portion of MD trajectories (100–300 ns), whose lengths are 2.5-fold bigger than those employed in the original training set; therefore, the high TPR% is unlikely due to correlation between trajectories in training/test sets. In line with this hypothesis, when states A are predicted from inhibitor-free trajectories of analogous length, we rather observed an overall decrease in specificity, indicating that, in contrast with its ability to recognize I states, the GDF-SVM fails to recognize A states when are taken from extended time windows and the model is trained on shorter trajectories. Overall, discriminative SVM models return more balanced predictions on our data when trajectories are of the same length during training and testing procedures (Figure 3g). In contrast, generative NB models are more robust to changes in the test sets, recovering interesting relationships between predicted percentage of states I and ligand functional properties, while keeping good specificity in recognizing patterns of the unperturbed TRAP1 states. When regression models are built by correlating the docking scores of the 8 or 11 compounds to their functional effects, the plots in Figure 3l,m show a regression line with a negative slope, indicating the lack of any correlation between the predicted score and their inhibitory power. In this respect, ML-based classification of inhibitors from MD simulations outperform docking-based models in the prediction of ligand-induced perturbation of TRAP1 dynamics and its connection to function.

## Discussion

Here, an ML approach was used to explore the existence of local dynamic patterns featuring inhibitor-free (A) and inhibitor-bound (I) TRAP1 states in a comparative MD ensemble including 66 systems, wherein 11 new allosteric modulators were used to train and validate NB and GDF-SVM models. These two different algorithms, based on probabilistic or nonprobabilistic approaches, were used in synergy with MD simulations of TRAP1 complexes to explore their ability to explain allosteric perturbation as a function of localized dynamic patterns developed on the ns−μs time scale. Such patterns were established as a particular combination of local dynamic features. In turn, the selection of such descriptors was guided by experimental results that demonstrated their role in modulating TRAP1 ATPase activity and in responding to nucleotide binding. On the basis of the hierarchy of time scales in protein motions,^6^ we hypothesized that allosteric perturbations could reverberate in changes of the dynamics at the level of inherently dynamic (local) segments, since the latter were known to drive the onset of slower functionally oriented conformational changes. Generative NB and discriminative SVM models were employed to learn from MD trajectories and to compare the performances of the two different approaches to the classification tasks. The two training sets were built based on retrospective data obtained on the three most active ligands. Consequently, the training sets were actually smaller than the test sets, where the activities of up to 8 or 11 ligands with lower or equal inhibitory efficacy were predicted and used to test performances on trajectories not included in the models. One of the hypotheses we tested was that the most active compounds would have been representative of local patterns inducing the maximum impact on local dynamics in the comparative ensemble, and that the less active ligands could have induced a nonoptimal perturbation of the set of functionally relevant local features, as compared to the most efficacious compounds. Generative models were the best ones with regard to validating this hypothesis. Linear regression analyses based on predicted TPR% for individual sets of inhibitor-bound systems returned models explaining from 64 to 71% of the variance expressed in the observed range of TRAP1 inhibition (Figure 3b,f). However, discriminative models did not reach similar correlations but consistently allowed to recognize opposite trends and generate boundaries between states. Therefore, GDF-SVM models provided a more qualitative distinction between the perturbed and the unperturbed ensembles without direct correlation to the experimental percentage of TRAP1 modulation, as shown by *r*^2^ values <0.60 (Figure 3g–i). These differences in performance suggested that NB models took advantage not only of the probabilistic treatment of the A/I states visited within the comparative MD ensemble but also of the assumption of independence among features. Even if allosteric motions act cooperatively on long time scales, such dependence could not be readily established on the simulated ones, but the local dynamic equilibrium of each feature can still independently respond to ligand perturbation to a different extent, depending on specific allosteric mechanisms and communication propensity of distal sites. Hence, the efficacy of an allosteric ligand may depend on the ability to interfere with an efficient combination of features. The Bayesian approach, weighting the ligand effects on each individual feature and treating them independently within a system-specific statistic model, enabled meaningful interpretation of puzzling details of allosteric propagation. We believe this makes NB an attractive model to analyze similar events in comparatively framed MD ensembles generated and simulated in the same conditions. In contrast, discriminative models, simply trying to separate the states based on the input *n*-dimensional space, do not take into account the probability that connects members of a class to the ensemble of visited features. This aspect probably induced the less active compounds to segregate from the unbound replicates by sampling specific regions which, however, were less representative of the patterns which instead were relevant to explain functional inhibition in our best inhibitors. By comparing predicted TPR% and FPR% in the 2D plots we showed that in the absence of ligands the unperturbed systems sample both patterns A and I, with the majority of systems exhibiting preference for state A. As for the global dynamic equilibrium regulating active/inactive pre-existing configurations, we did not exclude that states A and I might coexist on a local scale. Consistent with the hierarchy of time scales in protein motions and the extended conformational selection model for allostery,^4,6^ the coexistence of opposite local patterns at responsive TRAP1 elements in a near-native energy minimum may locally initiate a dynamic change that results in a global population shift in a more efficient way. Alternatively, one of them could be simply stabilized or destabilized as a consequence of allosteric perturbation and as a function of the ligand mechanism of action. In this respect, NB models are built so that conditional probabilities of features in a given class could be extracted and the weight of a feature quantified in each state. This property enables the identification of individual or combined contribution of the features to each classified MD frame and could assist further integrative biology studies to rationalize the diverse nature of allosteric modulations or, more widely, perturbing mechanisms. From the drug discovery standpoint, we showed that by building and validating balanced NB models on the most active ligands of a known series the models could learn patterns which generalize on the behavior of a much larger ensemble of completely “unseen” trajectories. The KNB model trained on the extended training set provided a useful interpretation of the degree of inhibitor-induced perturbation of the local functional dynamics and was retrospectively validated by achievement of meaningful correlation (0.71) between TPR% and percentages of TRAP1 inhibition. By relying on this model, compounds showing weaker inhibition of chaperone function could have been filtered out from the set, without losing the most promising hits and avoiding experimental testing.

## Conclusion and Perspectives

By applying our strategy to a real-world example, we highlighted the interesting potential of ML in maximizing the information contained in (chaotic but) easy-to-access MD simulations in the ns−μs time scale. Here, experimentally guided selection of local functional features coupled to the choice of a proper analysis framework enhanced the identification of significant trends within a carefully built set of perturbed and unperturbed ensembles of states. The generated ML models were retrospectively validated on ligands having of inhibitory efficacy equal (large test set) or lower (small test set) than those used for training. In summary, our results suggest that ML-driven interpretation of local dynamics in a complex system could be transformed into novel knowledge that can be aptly exploited for mechanistic or hit-to-lead optimization studies. In the latter case, we envision perspective applications of ML analysis to fast MD simulations to generate comparative ensembles able to discriminate and predict the functional effect of allosteric ligands on a given target, thus complementing docking affinity data.

## Supporting Information Available

Methods; probability distributions for the eight features in TRAP1 states (PDF)
