# Supplementary material for: Machine Learning of Allosteric Effects: The Analysis of Ligand-Induced Dynamics to Predict Functional Effects in TRAP1
Source: J Phys Chem B. 2020 Dec 28;125(1):101–14. doi: 10.1021/acs.jpcb.0c09742 (PMC8016192; doi:10.1021/acs.jpcb.0c09742)
Supplement: Supplementary file 1 — jp0c09742_si_001.pdf [file jp0c09742_si_001.pdf]

Supporting Information

Machine Learning of Allosteric Effects: The Analysis of Ligand Induced Dynamics to Predict Functional Effects in TRAP1

Mariarosaria Ferraro,[1] Elisabetta Moroni,[1] Emiliano Ippoliti,[2,3] Silvia Rinaldi,[1] Carlos Sanchez-Martin,[4] Andrea Rasola,[4] Luca F. Pavarino,[5,*] Giorgio Colombo[1, 6,*]

[1] Istituto di Scienze e Tecnologie Chimiche "Giulio Natta"– SCITEC, Via Mario Bianco 9, 20131 Milano, Italy.

[2] Institute for Advanced Simulation (IAS-5) and Institute of Neuroscience and Medicine (INM-9), Computational Biomedicine, Forschungszentrum Jülich, 52425 Jülich, Germany.

[3] JARA-HPC, Forschungszentrum Jülich, D-54245 Jülich, Germany.

[4]Dipartimento di Scienze Biomediche, Università di Padova, viale G. Colombo 3, 35131 Padova, Italy

[5]Dipartimento di Matematica “F. Casorati”, Università di Pavia, Via Ferrata, 5, 27100 Pavia Italy. Mail: luca.pavarino@unipv.it Phone: +39 0382 985643

[6] Dipartimento di Chimica, Università di Pavia, via Taramelli 12, 27100 Pavia, Italy. Mail: g.colombo@unipv.it Phone: +39 0382 987044

*Authors to whom correspondence should be addressed

Supplementary Methods

Basic Principles of Naïve Bayesian (NB) Algorithm

For generative Naïve Bayesian algorithms, maximum a posteriori probability (MAP) is employed as a decision rule for classification, holding on the statistical “naïve” assumption of features independence. This latter is applied to the Bayes’s theorem to verify whether state A or I is the most probable output, given a certain set of descriptors. This latter is the bayesian definition of the posterior (conditional) probability, $p(C_k|\, x_i)$, that the class exist as a specific features combination. Given the naive assumption, the posterior probability for a

class can be calculated *via* the product of the independent probabilities (joint probability) of each *i*-th feature of the vector *x* in a given class $p(x_i\,|C_k)$, which is in turn multiplied for the a priori probability $p(C_k)$ of each class in the training set.

Given *k* classes C_k, identified by inhibitor-bound (class I) and inhibitor-unbound (class A) states, a vector *x* of *i* features will be assigned to a label *y* = C_k according to the MAP calculated as in Eq. 1:

$$y = C_k = \underset{k\,\in\,\{I,A\}}{argmax}\, p(C_k)\prod_{i=1}^{8} p(x_i \mid C_k)$$

(1)

Basic principles of Support Vector Machines based on Gaussian Distribution Function (GDF-SVM)

Gaussian kernel functions are used in this SVM implementation as similarity functions to distinguish close from far features vectors in the mapped space. The criterion is established *via* a bell-shaped function *K* which associates to each pair of vectors, *x* and *l*, their distance in the kernel space (Eq. 2). Iteratively, the algorithm generates a gaussian curve centered

on vector *l*, defined as the landmark in the data set, ensuring that each *x* vector is treated as a landmark and viceversa in the training set. Each pair of vectors are then mapped as described below:

$$K(x,l) = exp\left(-\frac{\|x-l\|^2}{\sigma^2}\right) \tag{2}$$

The similarity between each landmark and its neighboring points decays with their radial distance and the decay rate is defined by the standard deviation of the curve, σ, which directly affect the decision boundary and the model resolution. Thereby, similar groups of vectors can be mapped onto the same class (low radial distances) in the space defined by the kernel function *K*. A linear decision boundary is then found to maximize the separation between classes. For non-separable classes, also a regularizing term C is used to learn the boundary. This is a penalty factor associated to misclassification. If C increases, the model tends to minimize misclassification reducing the maximum margin between classes, while lower values maximize the margin at the expense of misclassification.

Figure S1.

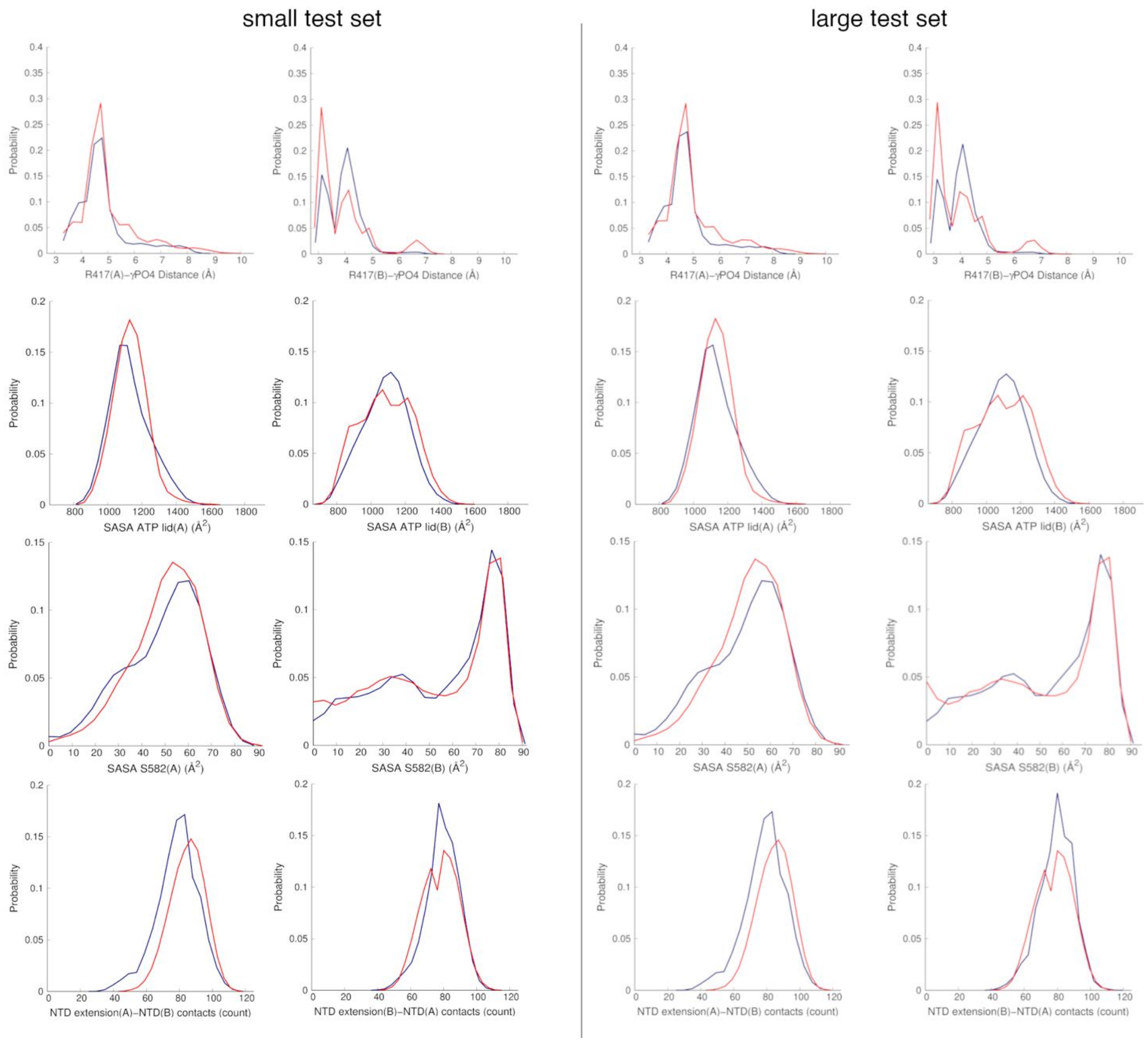

Figure S1. Probability distributions for the eight features in TRAP1 states A (blue) and I (red) in the small (1.92 µs for each state A/I) and large test sets (2.52 µs for each state A/I). The plots were obtained distributing individual features vectors collected from 24 inhibitor-unbound replicates and 24 inhibitor-bound complexes for the small test set. 3 inhibitor-bound and 3 inhibitor-free systems were added to the small test set to get distributions featuring the large test set (see **Table 1**).
